# Building Community Health Literacy to Achieve Health Equity: Insights from Ethiopian Tewahedo Social Services Community Leader in a County-Level Health Literacy Initiative

**DOI:** 10.1089/heq.2023.0069

**Published:** 2023-09-13

**Authors:** Ram Upreti, Kara Saiki, Mary Ann Abrams, Alexandria Jones, Krizia Melendez, Jessica Chevrolet, Heather Pennington, Aaron Leadingham, Denise Martin, Tetine Sentell

**Affiliations:** ^1^ Ethiopian Tewahedo Social Services, Columbus, Ohio, USA.; ^2^ Office of Public Health Studies, Thompson School of Social Work & Public Health, University of Hawai‘i at Mānoa, Honolulu, Hawaii, USA.; ^3^ Center for Child Health Equity and Outcomes Research, Nationwide Children's Hospital, Columbus, Ohio, USA.; ^4^ Department of Pediatrics, The Ohio State University College of Medicine, Columbus, Ohio, USA.; ^5^ Franklin County Public Health, Columbus, Ohio, USA.; ^6^ Columbus Public Health, Columbus, Ohio, USA.; ^7^ Nationwide Children's Hospital, Columbus, Ohio, USA.

**Keywords:** health literacy, community, stakeholders, public health, Bhutanese Nepalis, New Americans

## Abstract

This perspectives article shares insights from a county-level project in Franklin County, Ohio, to build collective organizational health literacy (HL) capacity across new sustainable networks to advance community-level HL. We provide an overview of the initiative followed by specific insights from a cultural liaison, the article's first author, who works in a community-based organization. He shares his collectivist perspective in building HL capacity at the grassroots level toward community-level goals. A shift in focus from individual responsibility to collective impact represents an important mindset change for attaining HL and builds on community strengths and values toward health equity.

## Introduction

The COVID-19 pandemic underscored the vital importance of health literacy (HL) across linguistic and cultural communities to combat misinformation, increase trust, and ensure health equity.^1^ Healthy People 2030 (HP 2030) includes HL among its foundational principles and overarching health equity goals.^2^ Importantly, HP 2030 articulates that HL is not an individual achievement, but a collective responsibility, highlighting the responsibility of organizations to “equitably enable individuals to find, understand, and use information and services to inform health-related decisions and actions for themselves and others”.^3^

Networks of organizations working together to achieve HL at the organizational and community level can build community-level HL.^4^ Community liaisons, including community health workers, within organizations are invaluable stakeholders in this process for engagement, trust, and patient-centered knowledge.^5^

This article, which arises from our project evaluation, provides key insights toward the goal of working in trusted multisectorial partnership in a county-level effort to build organizational HL capacity and sustainable networks to achieve community-level HL. We particularly highlight the perspectives of a key community liaison (R.U.) who worked in a trusted community-based organization (CBO) under a model of “Task Shifting” of some roles from health professionals into community capacity. This project exemplifies best practices and a deep ethos around health equity from a collectivist and capacity-building lens. We briefly describe the overall HL effort, share these perspectives, then consider implications for health equity.

## Advancing Health Literacy Franklin County

*Advancing Health Literacy Franklin County* (AHLFC) is a 2-year project funded by the Office of Minority Health begun in June 2021 focusing on organizational HL improvement, networks, and community engagement within Franklin County, Ohio, to build a health literate community.^6^ A health care organization (Nationwide Children's Hospital) collaborated with two public health agencies (Franklin County Public Health and Columbus Public Health), and CBOs to implement and establish culturally relevant HL-informed approaches to COVID-19–related communication and health system practices.

**FIG. 1.**
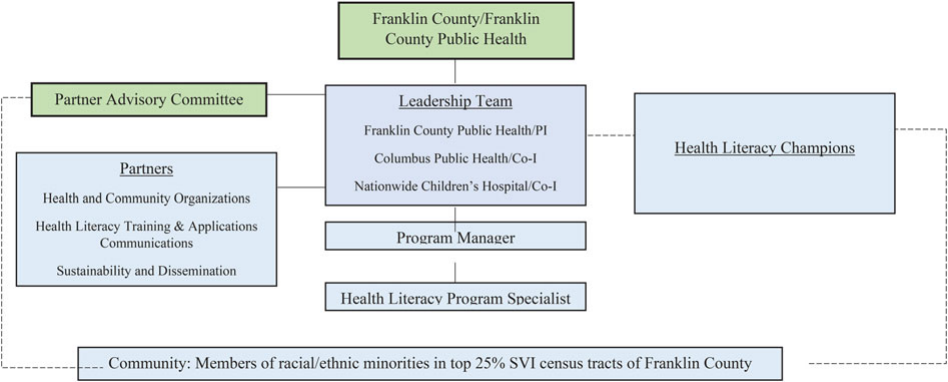
Advancing Health Literacy Franklin County Project organizational structure.

Franklin County Public Health provided project leadership and grant management, convened partners, and provided financial resources, communication, and data tools to the greater team. Columbus Public Health provided HL education and training to assist with organizational capacity building. Nationwide Children's Hospital provided content expertise, and capacity building and infrastructure development through a HL learning collaborative to establish HL expertise and embed HL-informed practices among diverse health-related organizations, and establishing a HL champion training program. Figure 1 provides an overview of the project's organizational structure.

The project identified racial and ethnic minority populations at highest risk for health disparities, low HL, and limited English proficiency not engaged through existing public health COVID-19 messages. Stakeholder engagement was critical to address this. In collaboration with CBOs serving these populations, the project worked to fill critical gaps in public health programming by learning from trusted community leaders and members to develop and share community-relevant HL-informed public health information. Table 1 provides insights into stakeholder engagement.

**Table 1.** Stakeholder Organizations and Project Partners to Franklin County at Inception, 2022

Partner	Role(s)	Responsibilities	Resources	Contributions
Resource partners				
Franklin County Public Health	Lead Partner, C, HO, SD	Project leadership and grant management; communications lead, data lead	Public health agency	Convener; messaging assessment; internal/external communication tools commns tools
University of Hawai'i at Mānoa	QI and Evaluation Lead	Lead equity-based evaluation and QI	MSI; research university	QI projects, DIS, evaluation reports
Columbus Public Health	HL, HO	HL Implementation Lead	Public health agency	HLHCO development; workforce development
Nationwide Children's Hospital	HL, HO	HL capacity-building; training lead; collaboratively implement HL applications	SME—HL; pediatric healthcare system	Comprehensive, tailorable tiered training menu and resources
Ethiopian Tewahedo Social Services	HL, CBO	New Americans Training Lead	SME—New Americans	CLAS-related training
Ohio Commission on Minority Health	SD	State partner	SME—minority health; state and national network	Policy recommendation sand dissemination
National Public Health Information Coalition	SD	National partner	SME—public health communications; national network	Policy recommendation sand dissemination
Health and community organizations				
National Council of Negro Women, Inc.	CBO	Role of HO/CBO Participate in project advisory group Team member completes HL training Participate in CLAS and other training and materials review Identify opportunities for improving, adding, and deploying messaging Participate in data collection, surveys, other Measurement and evaluation	Empower women of African descent	Contributions of HO/CBO partners Community engagement and voices HL workforce development PDSA message testing Balanced impact assessment
Partnership4Success	CBO		Youth services	
Our Lady of Guadalupe Center	CBO		Social service to reduce poverty	
National Medical Association (Columbus Ohio Chapter)	HO		African American physicians	
Ohio State Wexner Medical Center	HO		Academic medical center/FQHC	
OSU James CCC—Center for Cancer Health Equity	HO		Comprehensive cancer center on health equity	
Ohio University	HO		University and teaching hospital	
Heart of Ohio Family Health	HO		FQHC system	
Equitas Health	HO		FQHC with LGBTQ+ care	
PrimaryOne Health	HO		FQHC system	
Charitable Healthcare Network	HO		Diverse types of free clinics	
Buckeye Health Plan	HO		Medicaid and Medicare Plans	

C, communications; CBO, community-based organization; CLAS, Culturally and Linguistically Appropriate Services; DIS, Disparity Impact Statement; FQHC, Federally Qualified Health Center; HL, health literacy training and applications; HLHCO, Health Literate Health Care Organization; HO, health organization; MSI, Minority Serving Institution; PDSA, Plan-Do-Study-Act; QI, Quality Improvement; SD, sustainability and dissemination; SME, Subject Matter Expert.

A priority population for addressing health disparities in the AHLFC project was New Americans, defined as recent U.S. immigrants or children of a recent U.S. immigrant. Within the Franklin County racial/ethnic minority population, ∼14% are New Americans, including the largest U.S. population of Bhutanese Nepalis.^7^ The county is also home to Somali, Ethiopian, Eritrean, Congolese, Burmese, and Latino/Hispanic populations, representing many cultures and languages.^8^

The project partnered with Ethiopian Tewahedo Social Services (ETSS) to reach these populations. ETSS, which began >20 years ago with focus on the Ethiopian refugee and immigrant community, currently provides services for families from >89 countries and cultures and works to increase awareness of these cultures and histories in central Ohio.^9^ ETSS staff are trusted community members. As part of the AHLFC collaboration, ETSS provided cultural competency training to health care organizations to provide information and insights relevant to immigrant communities they serve, and ETSS team members received training in HL best practices, particularly teach-back, that can be valuable tools in their health outreach.

Teach-back is “a way of checking understanding by asking patients to state in their own words what they need to know or do about their health.”^10^ It allows confirmation that information has been shared in a way that patients understand.^10^

These efforts are part of ETSS's “Task Shifting” strategy in tackling wellness challenges among New American communities.^11^ The “Task Shifting” strategy is a community-based HL model wherein a trusted community liaison is trained in basic Western health prevention strategies and then some tasks that would be provided by a trained clinician/public health worker shift to the community liaison who can provide culturally and linguistically responsive services to their own community. The community liaison is a bridge across cultures that interprets language and culture. Cultural interpretation is more complex than language interpretation or translation.

The cultural liaison deeply knows the community norms, social life, structure, religion, art, culture, and language and interpretation of those terms in the contexts of their own community. The cultural liaison also knows the other culture by training and lived experience, so they can become a liaison in messaging for both cultures in a meaningful way or understanding. For example, depression has different meanings in different cultures than the Western medicine definition of depression. In some cultures, there is no word for depression.

A cultural liaison is the middle messenger between two cultures and makes a bridge between both easing communication barriers. A cultural liaison's goal is bridging of values of the culture of one community to another and connecting programs and services to culturally and linguistically diverse populations.

## Perspectives of ETSS Community Engagement and Career Coordinator

An ETSS Community Engagement and Career Coordinator has been engaging members of the Bhutanese Nepali and other neighboring communities throughout the pandemic. His perspectives highlight how he worked from—and beyond—his organizational role to build community HL.

*At the beginning of the pandemic, there was panic. Community elders were passing away from a virus that no one knew anything about. Other members of the community were worried about where and how to get the vaccine. I went door-to-door and left fliers with information. If people were not comfortable reading, I spoke with them about what vaccines were, their side effects, and why we need them. I even went on a Nepali radio show to share information, as well as through social media platforms like Facebook and TikTok. I was one of the first 10 persons in my office to have the vaccine and could be an example for my community.*

*For those who were hesitant or did not want to get vaccinated, I told them that they would not spread the virus to others and be protecting themselves and others around them. I did this day and night and referred ∼600 people through word of mouth and fliers, and as a result, 1167 people were vaccinated in Franklin county set up/scheduled clinics. We did interpretation for >250 clients at local clinics.*

*HL is about health, and how people are safe during this pandemic; how people can share information with their children and their neighbors and their friends. Because, once I tell them about what COVID is and what is the effect, and how it is going to spread from one to another, or when they get fliers about this, the impact multiplies. That is how people get reached.*

*It was not only one person; it went through one person—through me to somebody else. The impact goes multiplying, goes into the community, and from that it can reach the maximum limit.*

*I learned this by doing. As a social-minded person, I learned from doing things. Learning through books is not enough. Learning through experience is critical. People are hearing from an experienced person; that is also a way of learning. For instance, when individuals come to register for a test or to receive the vaccine, they may not know there are several medical questions. People may not understand because the words are difficult or because they do not speak the language. I also did not know those medical terms initially.*

*After I did several clinics, I asked questions. I worked with a staff member to understand these terms and then, as I registered people to get vaccinated at local clinics, I was able to interpret for them in the clinics and on the phone. I knew the medical terms and so it was easy for me to interpret to these people, to my people. It was also easy for me to relate to the people and to the staff who came to the clinic and to translate or interpret with the people for those difficult words and people could understand.*

*I always refer the people who cannot come to the clinics, and I talk to them. These people, some of my friends, they live near to my house, and I talk to them about how we can get vaccinated and address the pandemic. This is how I communicate with people wherever I meet them, not just in the clinics. I talk about how we can save our elders and how we can teach our younger generation. I felt so happy because I could help these people and let me volunteer for my community. This experience has shown the importance of having a willingness to serve your community in whatever way is needed and to be prepared to overcome unseen challenges in the field, with safety and community impact as priorities.*

## Discussion

The interactive collectivist perspective is critical to HL and health equity goals, as shown in the project overview and the perspective of the ETSS Community Engagement and Career Coordinator. Such insights highlight best practices in building networks of engagement and community HL specifically and in public health outreach generally, including the importance of social networks; perspectives beyond the individual, emphasizing community and family, including elders and children; building community HL efforts by supporting existing grassroots organizations and community relationships, as these cannot be readily created but can be supported; and conversation to confirm understanding and engagement (i.e., teach-back).^4,10–14^

As we move to sustain the overall project, the foundational capacity building and infrastructure development achieved—including training in teach-back and other HL-informed practices among community-based, public health, and clinical settings at the organizational level—will support those in cultural liaison roles, expand to address non-COVID-19 health disparities, and continue reaching priority populations. Coupled with ongoing impact through community engagement, partnerships, and organizational outreach and liaison personnel, this will further the collectivist perspective.

The ETSS Community Engagement and Career Coordinator's perspectives also demonstrate his incredible commitment, shared by so many individuals who work as cultural liaisons in their professional and personal lives.^11,15–17^ A shift in focus from individual responsibility to collective impact represents an important mindset change for attaining HL and builds on community strengths and values toward health equity. This shift also has the potential to amplify other health equity efforts tied to HL, like improving access to care and eliminating barriers to distribution of health information, as a collective perspective can have a greater impact on community through both collective engagement of individuals in social networks and trusted engagement with and across organizational partnerships.

Finally, we highlight the crucial importance of groundwork, cross-learning, and relationship building to achieve sustainable efforts for health equity at the community level that includes trust and longstanding partnerships. These may result in many outcomes that may be only visible over time or in the background of other successful initiatives (e.g., can be called upon in a crisis). Funds and policies that support this formative work, including support for the time and infrastructure of grassroots partners, are important areas for structural changes, as are developing evaluations that can account for the value and importance of these long-term relationships for equitable health outcomes even in project funding that may have a shorter time horizon.

## Abbreviations Used

AHLFC: Advancing Health Literacy Franklin County
CBO: community-based organization
ETSS: Ethiopian Tewahedo Social Services
HL: health literacy
HP 2030: Healthy People 2030
